# Preprocedural Substrate Visualization and Image Integration Based on Late Enhancement Computed Tomography for Ventricular Tachycardia Ablation in Non-Ischemic Cardiomyopathy

**DOI:** 10.3390/jcm14165801

**Published:** 2025-08-16

**Authors:** Jan-Hendrik van den Bruck, Jan-Hendrik Schipper, Katharina Seuthe, Sebastian Dittrich, Theodoros Maximidou, Arian Sultan, Jana Ackmann, Jonas Wörmann, Cornelia Scheurlen, Jakob Lüker, Daniel Steven

**Affiliations:** 1 Department for Electrophysiology, Heart Center University Hospital of Cologne, 50937 Cologne, Germany; jan-hendrik.schipper@uk-koeln.de (J.-H.S.); katharina.seuthe@uk-koeln.de (K.S.); sebastian.dittrich1@uk-koeln.de (S.D.); theodoros.maximidou1@uk-koeln.de (T.M.); jonas.woermann@uk-koeln.de (J.W.); cornelia.scheurlen@uk-koeln.de (C.S.); jakob.lueker@uk-koeln.de (J.L.); daniel.steven@uk-koeln.de (D.S.); 2 St. Georg Heart Center Hamburg, Asklepios Clinic Hamburg, 20099 Hamburg, Germany; epinhamburg@gmail.com

**Keywords:** ventricular tachycardia, inHEART, late iodine enhancement CT, preprocedural substrate visualization

## Abstract

**Background/Objectives**: Catheter ablation is an established therapy for ventricular tachycardia (VT), though outcomes remain limited in patients with non-ischemic dilated cardiomyopathy (NIDCM) due to complex arrhythmogenic substrates. Late iodine enhancement computed tomography (LIE-CT) offers a promising alternative to cardiac MRI for preprocedural substrate visualization. This study evaluated procedural characteristics and outcomes of LIE-CT-supported VT ablation versus conventional mapping (CM) in NIDCM patients. **Methods**: NIDCM patients undergoing VT ablation between January 2022 and August 2024 were retrospectively analyzed. LIE-CT data were processed using inHEART software. Patients were matched 1:1 by propensity score based on baseline characteristics, electrical storm, and prior ablations. **Results**: A total of 46 patients (mean age 59 ± 16.4 years, 74% male) were included (23 LIE-CT, 23 CM). Procedure durations were comparable (231.5 ± 74.2 vs. 220.2 ± 70.2 min, *p* = 0.5), but mapping time (35.9 ± 15.3 vs. 54 ± 5 min, *p* < 0.001) and fluoroscopy time (14.7 ± 5.1 vs. 21.3 ± 10.6 min, *p* = 0.02) were significantly shorter with LIE-CT. Epicardial access was more frequent (52% vs. 26%, *p* < 0.001), and bipolar ablation for intramural scar was performed in 17% of LIE-CT cases. There were no significant differences in acute kidney injury or 30-day mortality. At a median follow-up of 367 days, VT-free survival was 57% with LIE-CT and 52% with CM (*p* = 0.8). **Conclusions**: LIE-CT-supported VT ablation and substrate visualization was safe, without additional risk of acute kidney injury, and enabled more efficient and targeted VT ablation. Prospective studies are warranted to assess its impact on long-term outcomes in NIDCM patients.

## 1. Introduction

Catheter ablation is a well-established treatment for ventricular tachycardia (VT); however, its effectiveness in patients with non-ischemic dilated cardiomyopathy (NIDCM) is limited by the complex nature of the underlying arrhythmogenic substrate [1,2]. In NIDCM, fibrotic substrate differs substantially from that observed in ischemic cardiomyopathy, often involving mid-myocardial and subepicardial regions, particularly around the basal perivalvular zones and interventricular septum [2,3,4]. These differences reduce the applicability of strategies developed for ischemic substrates and are reflected in less favorable outcomes and higher VT recurrence rates in NIDCM patients, especially following endocardial-only ablation [2]. 

Although electroanatomic mapping (EAM) remains the gold standard for substrate identification during VT ablation, it has notable limitations, including the inability to detect intramural substrate and to reliably differentiate epicardial fat from scar tissue [5]. In this context, late gadolinium enhancement cardiac magnetic resonance imaging (LGE-CMR) has been widely employed for tissue characterization and scar localization. However, many patients with VT carry implantable cardiac electronic devices, which may preclude CMR or significantly impair image quality due to artifact [5].

Given the similar contrast kinetics of gadolinium and iodinated contrast agents, late iodine enhancement cardiac computed tomography (LIE-CT) has emerged as a promising alternative for noninvasive scar imaging [6,7]. Nevertheless, evidence on LIE-CT-supported VT ablation remains limited, particularly in patients with NIDCM. The present study aimed to evaluate procedural characteristics and outcomes of LIE-CT supported VT ablation in NIDCM patients, compared to a matched cohort undergoing ablation with conventional mapping techniques.

## 2. Materials and Methods

This single-center retrospective analysis included consecutive patients with NIDCM who underwent LIE-CT-supported VT ablation between January 2022 and August 2024. Patients were matched 1:1 using a propensity score (out of 83 patients) based on baseline characteristics, presence of electrical storm, and number of prior VT ablations, using data from a dedicated REDCap database (Nashville, TN, USA). The study was approved by the institutional review board (17-440, 14 December 2017), and informed consent was obtained from all patients.

### 2.1. Conventional Mapping

Substrate and/or activation maps were created using CARTO3^®^ (Johnson & Johnson MedTech, New Brunswick, NJ, USA) or EnSite™ X (Abbott, Chicago, IL, USA). High-density mapping was performed in all cases using a multipolar catheter (PentaRay^®^ Johnson & Johnson MedTech, New Brunswick, NJ, USA or Advisor™ HD-Grid Abbott, Chicago, IL, USA) [8,9]. For substrate mapping, bipolar electrogram amplitudes <0.5 mV were classified as dense scar, and values between 0.5 and 1.5 mV as border zone. Abnormal electrograms, including local abnormal ventricular activities, fractionated signals, and late potentials, were annotated. VT mapping identified ablation targets based on diastolic potentials, a post-pacing interval within 30 ms of the VT cycle length after entrainment, and/or localization of the critical isthmus via activation mapping [10].

### 2.2. inHEART Imaging and Image Integration

**Figure 1:**
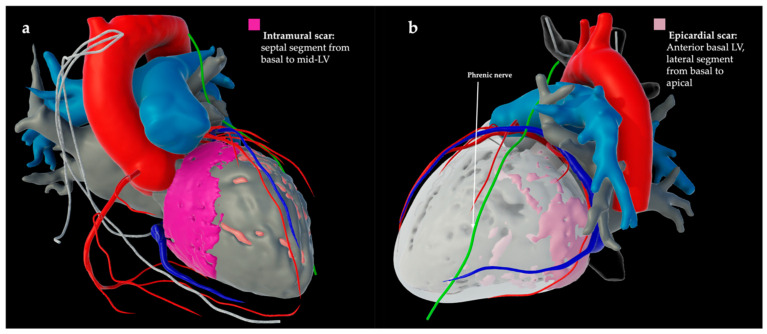
Late iodine enhancement CT (LIE-CT)-derived cardiac model after segmentation (inHEART, Pessac, France) of two patients with non-ischemic dilated cardiomyopathy. (**a**) Late iodine CT shows intramural scar (pink) in the septal left ventricle (LV) from basal to mid-LV. (**b**) Late iodine CT shows epicardial scar (light pink) on the anterior basal LV and the lateral segment from basal to apical.

For LIE-CT-supported VT ablation, contrast-enhanced, ECG-gated CT scans with delayed imaging (7–15 min post contrast) were acquired 2–5 days prior to ablation using 150 mL of iodinated contrast agent, and subsequently processed using dedicated software (inHEART Model Shaper v1.1.1; inHEART 33600 Pessac France) [11,12]. The software enables segmentation of cardiac structures—including chambers, endocardium, great vessels, coronary arteries, epicardial fat, and the phrenic nerve—as well as wall thinning and perfusion features with late iodine enhancement to assess myocardial architecture. Areas of scar and fibrosis are identified based on delayed contrast retention [6,7]. An artificial intelligence-driven segmentation algorithm generates interactive 3D models, which are anonymized, uploaded to a secure platform, and integrated into electroanatomic mapping systems to support intraprocedural guidance (Figure 1) [11,13]. For 3D model integration, an additional conventional electroanatomic map was acquired in all patients and registered to the CT segmentation using anatomical landmarks such as the aortic root, pulmonary veins, and coronary sinus.

### 2.3. VT Ablation

In all patients, programmed ventricular stimulation was performed at the beginning of the procedure to induce VT. Based on VT inducibility and hemodynamic tolerance, the ablation strategy was either substrate-based or performed during VT. Substrate modification targeted areas with low-amplitude fractionated electrograms, long stimulus-to-QRS intervals, late potentials, or optimal pace map sites. Lesion quality was assessed by targeting non-excitability, confirmed by loss of pace capture during or immediately after RF application. All procedures were performed using contact force-sensing catheters, with an impedance drop of ≥10 ohms defined as the target for effective lesion formation [10,14]. The primary procedural endpoint was non-inducibility of any VT following programmed right ventricular stimulation at the end of the procedure.

### 2.4. Periprocedural Acute Kidney Injury

Serum creatinine was measured following both contrast-enhanced CT acquisition and VT ablation. Acute kidney injury (AKI) was defined according to the KDIGO criteria as an absolute increase in serum creatinine of ≥0.3 mg/dL within 48 h or a relative increase of >1.5 times the baseline value [15]. Periprocedural AKI was defined as cumulative renal impairment occurring within the periprocedural period, encompassing both the procedural risk of VT ablation and the additional contrast load from CT imaging. Chronic kidney disease (CKD) was defined as a baseline estimated glomerular filtration rate (eGFR) of <60 mL/min/1.73 m^2^ [16].

### 2.5. Statistical Analysis

Data analysis was conducted using SPSS Statistics (Version 27, IBM, Chicago, IL, USA). Categorical variables are presented as numbers and percentages, and continuous variables as mean with standard deviation or median with interquartile range, as appropriate. Group comparisons for categorical variables were performed using the chi-square or Fisher’s exact test, and continuous variables were compared using Student’s *t*-test or the Mann–Whitney U test. Propensity scores were calculated based on the following covariates: age, body mass index, sex, left ventricular ejection fraction, presence of electrical storm, and number of prior VT ablations. Matching was performed using a nearest-neighbor algorithm with a caliper of one-fifth of the standard deviation. Each patient in the LIE-CT group was matched 1:1 with a patient from the CM group.

## 3. Results

### 3.1. Patient Characteristics

A total of 46 patients with NIDCM were included in this analysis: 23 patients underwent LIE-CT supported VT ablation (age 60.1 ± 11.4 years, 74% male) and 23 underwent CM VT ablation (age 60.4 ± 11.6 years, 74% male). The groups were well balanced, and detailed patient characteristics are presented in Table 1. An implantable cardioverter defibrillator (ICD) was present in 96% of patients, with single-chamber ICDs and cardiac resynchronization therapy–defibrillators (CRT-Ds) being the most common device types. Eleven patients (48%) in each group had experienced an electrical storm prior to ablation, and 11 patients per group had no history of previous VT ablation.

### 3.2. Procedural Characteristics

All procedures were performed under deep sedation with ultrasound-guided vascular access. Programmed right ventricular stimulation (S1/S2/S3) was conducted in all patients for VT induction; 78% of the LIE-CT group and 87% of the CM group (*p* = 0.4) were inducible for VT. Although overall procedure duration was comparable between groups (231.5 ± 74.2 vs. 220.2 ± 70.2 min, *p* = 0.5), the LIE-CT group demonstrated significantly shorter mapping time (35.9 ± 15.3 vs. 54.5 ± 14.2 min, *p* < 0.001), reduced fluoroscopy duration (14.7 ± 5.1 vs. 21.3 ± 10.6 min, *p* = 0.02), and lower fluoroscopy dose (1278.9 ± 1224.4 cGy·cm^2^ vs. 4444.1 ± 2842.1 cGy·cm^2^, *p* < 0.001), but longer RF application time (42.4 ± 17.9 vs. 27.9 ± 14.2 min, *p* = 0.008). While the overall ablation strategies were similar (Table 2), epicardial access was more frequently performed in the LIE-CT group (52% vs. 22%, *p* = 0.03), and bipolar ablation—used to treat intramural substrate identified via LIE-CT—was applied exclusively in this group (22%, *p* = 0.02). Detailed procedural characteristics are presented in Table 2.

### 3.3. Acute Outcome and Discharge

Non-inducibility of any VT was achieved in 61% of patients in the LIE-CT group and in 53% in the CM group (*p* = 0.6). Clinical VT remained inducible in a small subset of patients in both groups (9% vs. 4%, *p* = 0.6). Post-procedural monitoring requirements were comparable (Table 3), with approximately half of the patients in each group admitted to intermediate care. Median hospital stay was 3 days (IQR 2–6) in both groups, regardless of mapping strategy.

### 3.4. Procedural Complications

Complications occurred in 4 out of 46 procedures, with two events in each group. Each group experienced one minor access site-related complication not requiring further intervention. In the LIE-CT group, one patient developed a coronary artery stenosis following bipolar ablation requiring immediate revascularization. In the CM group, one patient developed third-degree atrioventricular block during septal ablation. No additional complications were reported. Despite the use of an additional preprocedural contrast-enhanced CT in the LIE-CT group, the incidence of periprocedural acute kidney injury was comparable between groups, occurring in three patients in the LIE-CT group and four in the CM group (*p* = 0.7).

### 3.5. Outcome and Mortality

**Figure 2:**
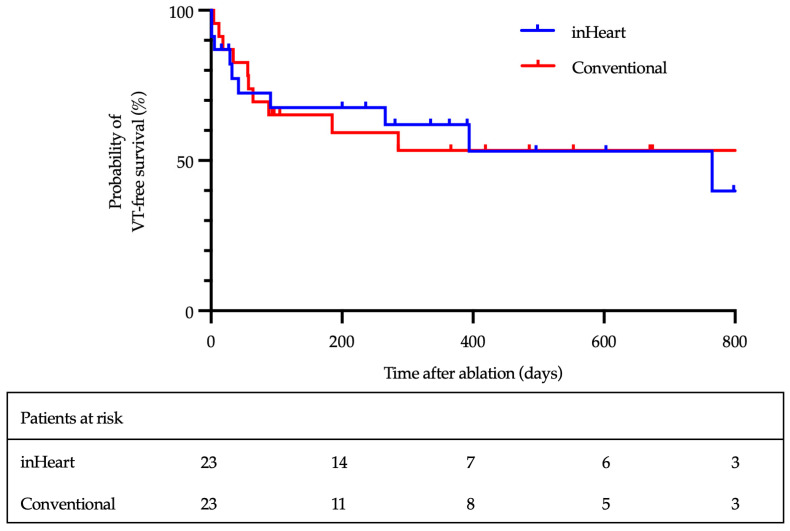
Kaplan–Meier analysis of VT-free survival.

After a median follow-up of 367 days (IQR 251–555), 13 of 23 patients (57%) in the LIE-CT group and 12 of 23 patients (52%) in the CM group remained free from VT recurrence (Figure 2; log-rank *p* = 0.8). Three patients in each group died from terminal heart failure after a median of 60 days (IQR 18–171). The 30-day mortality rate was 6%.

## 4. Discussion

VT ablation in patients with NIDCM remains one of the most complex and challenging procedures in interventional electrophysiology. Despite advancements in 3D mapping technologies and catheter design, outcomes in this patient population remain suboptimal. Therefore, novel mapping and ablation strategies are needed to enhance individual substrate characterization and ultimately improve procedural success and clinical outcomes. Currently, data on LIE-CT-supported VT ablation in NIDCM is scarce. In this context, the present analysis offers several important findings:

- Preprocedural contrast-enhanced CT was safely performed without increasing the risk of periprocedural acute kidney injury.
- The LIE-CT group had shorter mapping times (*p* < 0.001) and reduced fluoroscopy duration (*p* = 0.02) and dose (*p* < 0.001), allowing for longer RF application times (*p* = 0.008).
- Substrate visualization was linked to more complex procedures, with higher rates of epicardial access (52% vs. 22%, *p* = 0.03) and bipolar ablation (22% vs. 0%, *p* = 0.02).
- In this cohort, in which nearly half of the patients had a history of electrical storm, additional substrate imaging did not result in improved clinical outcomes—highlighting the ongoing challenges in managing VT in non-ischemic cardiomyopathy.

### 4.1. Safety

The overall major complication rate (9%) was low, especially given the complexity of the procedures, and aligns with previously reported rates in VT ablation for structural heart disease [15]. In the LIE-CT group, one patient developed a left anterior descending (LAD) coronary artery stenosis requiring urgent revascularization after bipolar ablation—a rare but recognized complication that highlights the need for careful anatomical evaluation when applying bipolar RF energy near coronary vessels [16]. In the CM group, one patient developed complete atrioventricular block during septal ablation. The 30-day mortality was 6%, with two patients in the LIE-CT group and one in the CM group dying from terminal heart failure during the index hospitalization; no procedure-related deaths occurred. Periprocedural acute kidney injury was observed in approximately 9% of patients, with no significant difference between groups (*p* = 0.7), consistent with previously reported rates in VT ablation cohorts [15].

### 4.2. Procedural Aspects

A key finding of this study is the significantly shorter mapping time and reduced fluoroscopy burden in the LIE-CT group, despite comparable overall procedure times. These results suggest that preprocedural anatomical and substrate imaging with LIE-CT may streamline the mapping process by facilitating more targeted catheter navigation and focused electroanatomic mapping. Compared to previously reported LIE-CT-guided ablation procedures in patients with ischemic cardiomyopathy, procedure durations in this study were longer, likely reflecting the greater complexity of VT ablation in the NICM population [17].

While Englert et al. did not report a reduction in fluoroscopy exposure in their VT ablation study using imaging guidance, our findings are the first to demonstrate such a benefit in VT ablation—aligning with earlier reports of reduced fluoroscopy time in atrial fibrillation ablation procedures integrating CT imaging [12,18].

The significantly longer radiofrequency (RF) application time in the LIE-CT group (42.4 vs. 27.9 min) likely reflects more targeted and extensive substrate modification guided by preprocedural imaging. Prior studies have shown that VT ablation in NIDCM often requires broader lesion sets due to the frequent presence of diffuse, intramural, or subepicardial scarring [19]. LIE-CT may enhance the ability to identify and localize these challenging substrates, allowing for more comprehensive ablation.

Furthermore, the greater frequency of epicardial access and the exclusive use of bipolar ablation in the LIE-CT group highlight the potential of advanced imaging to inform procedural strategy.

Intramural and subepicardial substrates—common in NIDCM—are often inaccessible by endocardial ablation alone [19]. Early identification of such substrates via LIE-CT may support the decision to pursue epicardial access up front, helping avoid unnecessary epicardial procedures in patients without epicardial scarring and thereby minimizing the associated complication risk, reported at 4–7% in previous studies [5]. Additionally, accurate localization of intramural substrates and integration of anatomical landmarks such as coronary arteries is essential for successful bipolar ablation, a technically demanding technique that relies heavily on precise anatomical orientation [20].

### 4.3. Outcome

Despite the use of advanced imaging for preprocedural substrate visualization, this study did not demonstrate a significant difference in VT recurrence between groups, with 57% of patients in the LIE-CT group and 52% in the CM group remaining free from VT at a median follow-up of 367 days (log-rank *p* = 0.3). Notably, this cohort included a high-risk population, with nearly half of the patients presenting with a history of electrical storm, known to be associated with increased arrhythmic burden and poorer outcomes [21].

These findings contrast with those reported by Englert et al., who observed improved arrhythmia-free survival in patients undergoing VT ablation guided by inHEART-based CT models [12]. Several factors may account for the differing outcomes between the two studies, including variations in patient populations, baseline VT burden, prior ablation history, sample size, and follow-up duration. Importantly, while Englert et al. reported no mortality, six patients in our cohort died from terminal heart failure, underscoring the high-risk profile of this population. Additionally, John et al. described a threefold increase in VT recurrence in patients with septal scar identified by LIE-CT, which may have further contributed to our observed outcomes [22]. These observations highlight that although LIE-CT integration offers procedural benefits, its consistent impact on long-term clinical outcomes in NIDCM remains uncertain and warrants further prospective investigation.

### 4.4. Cost-Effectiveness

The use of LIE-CT involves additional costs and risks related to scanner time, contrast administration, and radiation exposure. However, in our cohort, it was associated with reduced intraprocedural radiation dose, shorter mapping duration, and improved anatomical visualization to guide advanced ablation strategies. Importantly, LIE-CT enables identification of critical structures such as the phrenic nerve and coronary arteries, potentially enhancing procedural safety. It can also help avoid unnecessary epicardial access or justify its use by confirming the absence or presence of epicardial substrate [5]. The cumulative reduction in fluoroscopy dose additionally benefits the operator by lowering occupational radiation exposure.

These advantages may justify the added resource use, particularly in this complex patient population with generally limited procedural success. However, prospective randomized trials are needed to determine whether these benefits translate into improved clinical outcomes and reduced complication rates.

### 4.5. Limitations

This study is a single-center, non-randomized, retrospective analysis with the inherent limitations of such a design.

Due to the specificity of the patient population, the overall sample size is limited, which restricts the use of more advanced statistical analyses. As a result, non-significant differences in VT-free survival may reflect insufficient statistical power rather than true equivalence. In addition, potential confounding related to differences in procedural strategy (e.g., more frequent epicardial access) or RF duration cannot be fully excluded.

Therefore, the findings should be interpreted as exploratory and hypothesis-generating.

To reduce selection bias, we included consecutive patients and applied propensity score matching. Nevertheless, a prospective, randomized trial is necessary to validate these findings and more definitively assess the impact of preprocedural substrate visualization on outcomes in this complex patient cohort.

## 5. Conclusions

LIE-CT-guided preprocedural substrate visualization was safely implemented without increasing the risk of contrast-induced kidney injury. Integration of the LIE-CT model led to shorter mapping times, reduced fluoroscopy exposure, and improved identification of complex intramural and epicardial scar, enabling both bipolar and epicardial ablation. Its consistent impact on long-term clinical outcomes in NIDCM remains uncertain and warrants further prospective, randomized investigation.

## Figures and Tables

**Table 1:** Patient characteristics.

	LIE-CT Group	CM Group	*p*-Value
Sex (male)	17 (74%)	17 (74%)	1.0
Age (years)	60.1 ± 11.4	60.4 ± 11.6	0.9
BMI (kg/m^2^)	28.9 ± 5.4	28.2 ± 5.2	0.7
LVEF (%)	35.4 ± 9.2	34.7 ± 9.5	0.8
GFR (mL/min)	60.9 ± 16.8	59.0 ± 24.0	0.8
Electrical storm	11 (48%)	11 (48%)	1.0
Prior VT ablations	1 (IQR 0–1)	1 (IQR 0–1)	0.9
No prior VT ablations	11 (48%)	11 (48)	1.0
**Cardiac implantable electronic devices**			
Patients without defibrillator	1 (4%)	1 (4%)	1.0
Single-chamber ICD	8 (35%)	8 (35%)	1.0
Dual-chamber ICD	2 (9%)	3 (13%)	0.6
CRT-D	8 (35%)	9 (39%)	0.8
S-ICD	4 (17%)	2 (9%)	0.4
**Comorbidities**			
Atrial fibrillation	9 (39%)	12 (52%)	0.4
Diabetes	5 (22%)	6 (26%)	0.7
Renal failure (GFR ≤ 60 mL/min)	6 (26%)	7 (30%)	0.7

**Table 2:** Procedural characteristics.

	LIE-CT Group	CM Group	*p*-Value
**Mapping System**			
EnsiteX	15 (65%)	14 (61%)	0.8
CARTO	8 (35%)	9 (39%)	0.8
**Access**			
Retrograde	4 (17%)	7 (30%)	0.3
Transseptal	10 (43%)	4 (17%)	0.06
Combined retrograde and transseptal	9 (39%)	12 (52%)	0.4
Epicardial access	12 (52%)	5 (22%)	0.03
**VT induction**			
Programmed RV stimulation	23 (100%)	23 (100%)	1.0
VT inducible	18 (78%)	20 (87%)	0.4
**VT ablation approach**			
Primarily substrate based (scar homogenization, LAVA)	11 (48%)	9 (39%)	0.6
Primarily ablation during VT	4 (17%)	7 (30%)	0.3
Combination of ablation during VT and substrate homogenization	8 (35%)	7 (30%)	0.8
VTs targeted per patient	1.8 ± 1.0	1.5 ± 0.8	0.3
Bipolar ablation	5 (22%)	0	0.02
**Procedure characteristics**			
Total RF time (min)	42.4 ± 17.9	27.9 ± 14.2	0.008
Fluoroscopy dose (cGy·cm^2^)	1278.9 ± 1224.4	4444.1 ± 2842.1	<0.001
Fluoroscopy duration (min)	14.7 ± 5.1	21.3 ± 10.6	0.02
Procedure duration (skin-to-skin; min)	231.5 ± 74.2	220.2 ± 70.2	0.5

**Table 3:** Acute outcomes and discharge.

	LIE-CT Group	CM Group	*p*-Value
**Acute Outcomes**			
Non-inducibility of any VT	14 (61%)	12 (53%)	0.6
Non-inducibility of clinical VT	2 (9%)	6 (26%)	0.1
Substrate based due to prior non-inducibility	5 (22%)	4 (17%)	0.7
Clinical VT still inducible	2 (9%)	1 (4%)	0.6
**Post-procedural monitoring**			
Intensive care	1 (4%)	2 (9%)	0.6
Intermediate care	12 (52%)	11 (48%)	0.8
Normal ward	10 (44%)	10 (43%)	1.0
**Periprocedural kidney injury**			
Acute kidney injury	3	4	0.7

## Data Availability

Data are available upon reasonable request made to the corresponding author.

## Abbreviations

The following abbreviations are used in this manuscript:

VT	Ventricular tachycardia
NIDCM	Non-ischemic dilated cardiomyopathy
LIE-CT	Late iodine enhancement computed tomography
CM	Conventional mapping
EAM	Electroanatomic mapping
AKI	Acute kidney injury
KDIGO	Kidney Disease: Improving Global Outcomes
CKD	Chronic kidney disease
BMI	Body mass index
LVEF	Left ventricular ejection fraction
GFR	Glomerular filtration rate
ICD	Implantable cardioverter defibrillator
CRT-D	Cardiac resynchronization therapy–defibrillator
S-ICD	Subcutaneous implantable cardioverter defibrillator
RF	Radiofrequency current
